# Anatomical relations of the superficial sensory branches of the radial nerve: a cadaveric study with clinical implications

**DOI:** 10.1186/1754-9493-5-28

**Published:** 2011-11-04

**Authors:** Lasitha B Samarakoon, Kasun C Lakmal, Sharmila Thillainathan, Vipula R Bataduwaarachchi, Dimonge J Anthony, Rohan W Jayasekara

**Affiliations:** 1 Department of Anatomy, Faculty of Medicine, University of Colombo, Sri Lanka

**Keywords:** SBRN, Cephalic vein, surgical incisions, venous cannulation

## Abstract

**Background:**

Anatomically, it is difficult to give a systematic description of the superficial branch of the radial nerve (SBRN). Our aim was to describe the exact relationship of the SBRN to fixed bony points of radial styloid and Lister's tubercle, and to the cephalic vein. We also compared our data with other international studies.

**Methods:**

The study was a descriptive anatomical study. Twenty-five forearms were dissected. Measurements were made from predefined fixed reference points.

**Results:**

The mean distance to the point of emergence of the nerve from the radial styloid was 8.54 cm (SD = 1.32). The nerve branched at a mean distance of 5.57 cm (SD = 1.43) from the radial styloid. The mean distance to the point where the most medial and most lateral branches of the nerve crossing the wrist joint, measured from the Lister's tubercle were 2.51 cm (SD = 0.53) and 3.90 cm (SD = 0.64). In 17 specimens(68%) cephalic vein crossed the SBRN superficially once. Mean distance from the radial styloid to the most distal point where the vein crossed the nerve was 5.10 cm. Diffefrence between mean distance to the point of emergence and branching point, when compared with other international studies were not statistically significant. (P value > 0.05)

**Conclusions:**

We recommend avoiding transverse incisions in the snuffbox region between 2.51 cm and 3.90 cm from the Listers tubercle. We also recommend avoiding cannulation of the cephalic vein in the distal forearm.

## Background

The superficial position of the sensory branch of the radial nerve (SBRN) is vulnerable to injury during a variety of surgical procedures including orthopedic percutaneous wire fixation, cephalic vein cannulation and arthroscopic surgery of the wrist joint.

In a study conducted by Glanvill, R. et al [1] the incidence of superficial radial nerve injury after Kirchner wire insertion by an experienced orthopedic surgeon was assessed. K-wires were inserted into the radii of 92 adult cadavers. Subsequent dissection of the area exposed the superficial radial nerve and any observed nerve injury was documented. It was concluded that nerve injury may still occur as a result of K-wire insertion.

In a similar cadaveric study conducted by Vandersluis, R. et al [2], risk of soft-tissue injury during percutaneous placement of external fixation pins in the proximal radius was assessed. It was noted that nerve or tendon injuries occurred in 7 of 26 forearms and interestingly, three pins transfixed either the superficial branch of the radial nerve or lateral antebrachial cutaneous nerves. Authors recommended open pin placement for fractures of the distal radius rather than percutaneous fixation as the risk of iatrogenic injury was significant.

Boeson, M. B et al [3] reported a case of a patient who had an intravenous catheter inserted into her cephalic vein and thereafter sustained an injury to the superficial branch of the radial nerve. Similar case reports of Superficial radial neuropathy caused by intravenous injection [4] and neuroma of the superficial branch of the radial nerve after intravenous cannulation [5] has been reported in the literature.

Iatrogenic injury to the SBRN during port insertion of arthroscopic surgery of the wrist joint was assessed in a cadaveric study by Kilic A et al [6]. Dissections were performed on 11 hands from 6 cadavers starting from the point where the SBRN begins to emerge and ending at the terminal branches of the dorsal hand. The distribution of the SBRN, the distance from the superficial branch to the dorsal portals used in wrist arthroscopy were studied. It was concluded that the limited size of the area where portals can be positioned and the anatomic variations between individuals are major obstacles in developing a guideline for reducing the risk of SBRN injury in wrist arthroscopy. Authors advised making a skin-only incision for this portal and then using blunt dissection to help prevent injury to the SBRN.

In a cadaveric study conducted by Abrams, R.A et al [7] twenty fresh human cadavers were dissected and anatomical relations of the superficial branch of the radial nerve was assessed with the aim of delineating its exact anatomy would help prevent injury during operative procedures on the radial side of the hand, wrist and forearm, as well as accurate localization in treatment of traumatic injuries or performance of nerve blocks in its distribution.

Adding to the above, in another study Robson, A.J et al [8] attempted to identify a safe incision for the release of de Quervain's tenosynovitis. It was noted that all 25 specimens had branches underlying the traditional transverse incision for de Quervain's release but a 2.5 cm longitudinal incision proximal from the RS avoided the SBRN in 17/25 cases (68%). It was concluded that A longitudinal incision in de Quervain's surgery may be preferable to the traditional transverse incision.

Similarly in another cadaveric study by Auerbach D.M. et al. [9], sixteen out of twenty specimens had branches directly overlying the typical transverse incision for De Quervain's release and 12 specimens had branches directly overlying the 3-4 wrist arthroscopy portal. Authors concluded that appreciation of the location of the superficial radial nerve and the proximity of branches to commonly used surgical incisions is important when performing surgical procedures over the dorsum of the hand and wrist.

Above studies amply illustrate the fact that anatomical variability and superficial course of the SBRN makes is very vulnerable to injury during a variety of surgical procedures. We have attempted in our study to clarify the nerves course and its branches in relation to fixed bony points so as to provide the surgeon with a "safe zone" for surgical incisions of the dorsum of the wrist so to minimize the risk of iatrogenic injury to the nerve. We also attempted to characterize the nerves course in relation to the cephalic nerve and to identify the points of intersection between the nerve and vein, as these are the points where there is highest risk of nerve injury during intravenous cannulation. By identifying these in relation to fixed bony points, which are easily accessible to the surgeon, we attempted to point out the surgical risks for an iatrogenic injury to the SBRN and how to minimize these during surgical procedures about the wrist.

## Methods

The study was a descriptive anatomical study with the aim of describing the exact relationship of the SBRN to fixed bony points, that of the radial styloid and the Lister's tubercle, as well as to describe the branching pattern and its relationship to cephalic vein. We attempted to identify potential anatomical landmarks in order to safeguard the nerve during surgical procedures that may otherwise place the nerve in danger.

25 forearms and wrists (10 left and 15 right) from cadavers of undetermined age and sex were selected. All of these cadavers of Sri Lankan ethnicity and were prepared using conventional arterial embalming technique using a solution containing formalin (as the main preservative) and carbolic acid and alcohol (as supporting solutions). Each specimen was carefully dissected, while preserving the tissue planes. First, a dorsolateral incision was made, from the elbow to the metacarpophalangeal joints. The cephalic vein was then identified and preserved. The sensory branch of the radial nerve was then dissected out as it emerged from under the brachioradialis muscle, at the upper third of the forearm. The nerve was then dissected distally to the emergence of its terminal branches.

**Figure 1:**
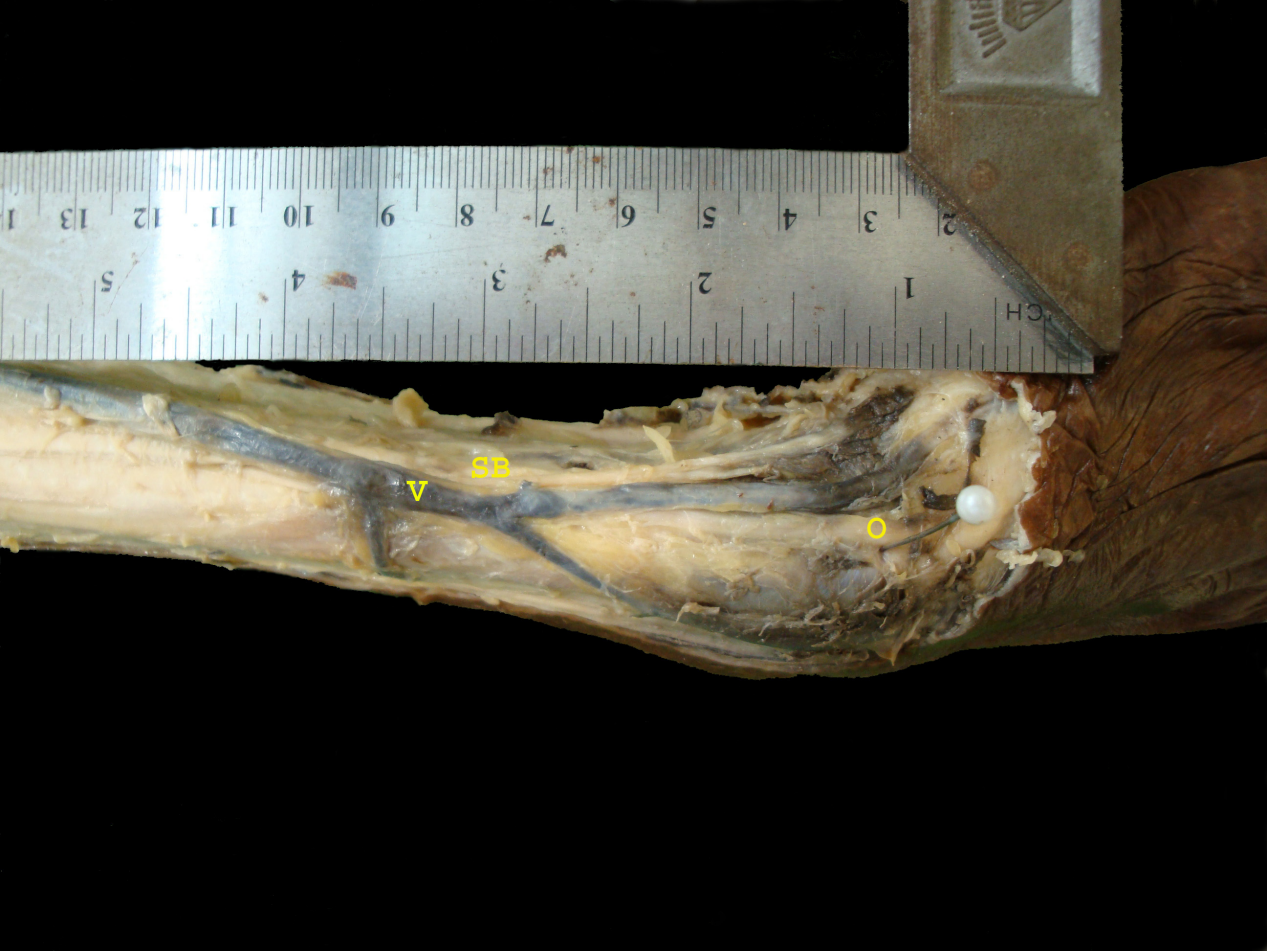
**A dissected specimen showing superficial branch of the radial nerve (SB), Cephalic vein (V), And the reference point-Radial styloid (O)**.

**Figure 2:**
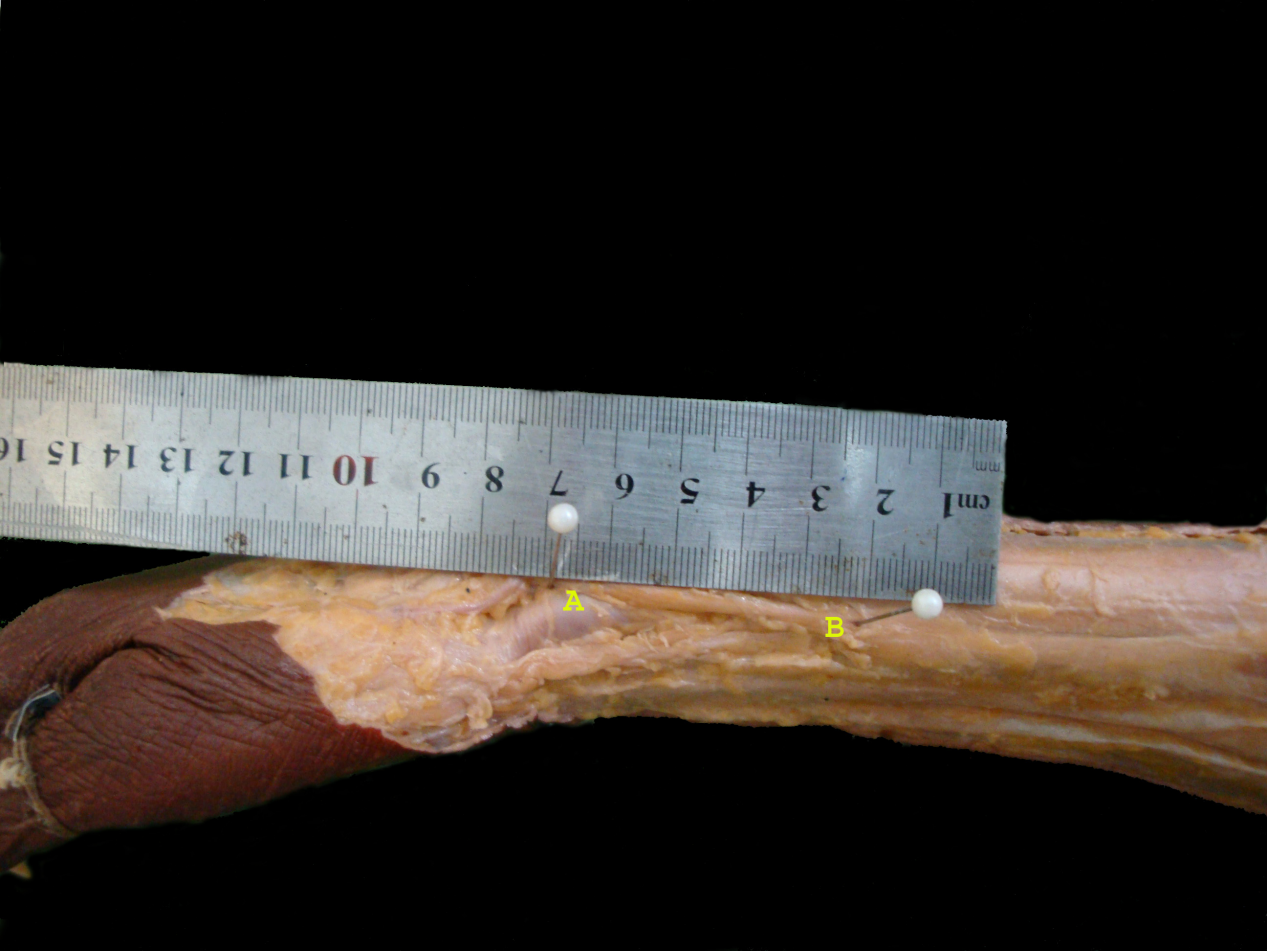
**A dissected specimen showing the point of emergence of the nerve (B), with the reference point-Radial styloid (A)**.

**Figure 3:**
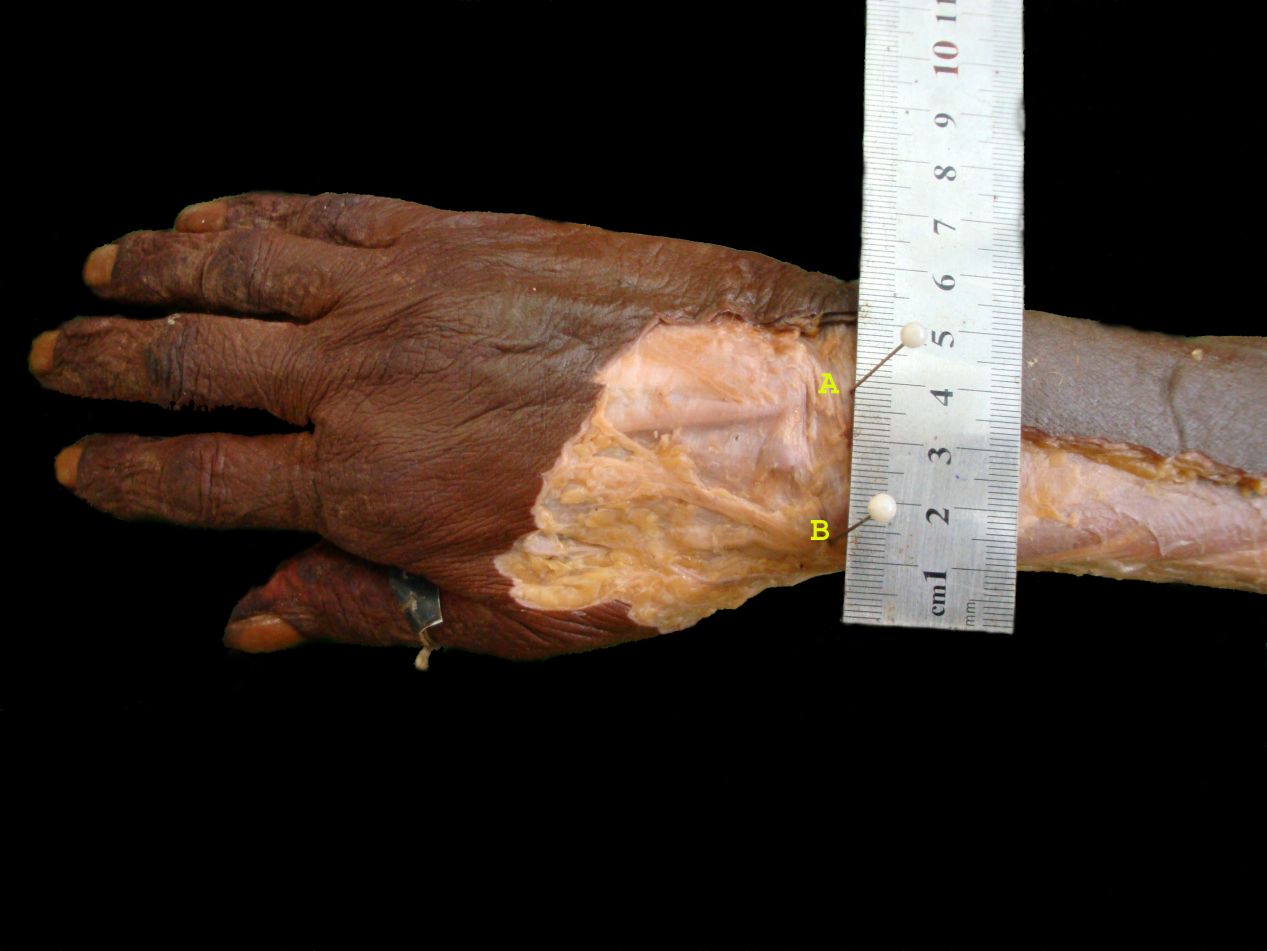
**A dissected specimen showing Listers tubercle (A) and the point where the nerve crosses the wrist at the level of the Listers tubercle (B)**.

The styloid process of the radius was defined as a reference point O (Figure 1). N was the point where the sensory branch of the radial nerve emerged between the brachioradialis muscle and the extensor carpi radialis longus tendon. Measurements were made along the axis of the forearm for each specimen (Figure 2). The ON distance represented the distance between the styloid process of the radius and the emergence of the nerve. N1 marked the point of division of the superficial radial nerve into a lateral branch that continued on the dorsal aspect of the thumb and a medial branch coursing on the dorsal aspect of the wrist. ON1 was the distance from the styloid process of the radius to this first division as measured along the axis of the forearm. N2 was defined as the point which the most medial branch of the nerve crossed dorsum of the wrist. N3 was defined as the point which the most lateral branch of the superficial nerve crossed the lateral aspect of the wrist and the radial styloid. Using Lister's tubercle of the radius as the reference point X, XN1 and XN2 distances were measured along a plane perpendicular to the axis of the forearm, at the level of the Lister's tubercle (Figure 3). V1 was the point where the cephalic vein crossed the superficial radial nerve. Additional intersections if observed were likewise named as V2, V3 etc. OV1 and OV2 distances were measured along the axis of the forearm between the styloid process of the radius and the V1 or V2 points, respectively. All distances were measured with the aid of a caliper.

All the specimens were dissected and measured in the lateral position, where the index fingers were lined up with the axis of the forearm, and the axes of the thumb maintained at a 45° angle with the index finger.

All data were analyzed using SPSS version 17 package. P values were calculated using students t test. P value < 0.05 was taken as statistically significant (CI 95%).

## Results

The mean distance to the point of emergence of the nerve beneath brachioradialis from the radial styloid was 8.54 cm (SD = 1.32) with a range from 5.10 cm to 11.14 cm.

The nerve branched constantly into 2 divisions in all 25 specimens, at a mean distance of 5.57 cm (SD = 1.43) from the radial styloid.

The mean distance to the point where the most medial and most lateral branches of the nerve crossing the wrist joint, measured from the Lister's tubercle were 2.51 cm (SD = 0.53) and 3.90 cm (SD = 0.64).

In 17 specimens (68%) the cephalic vein crossed the SBRN superficially once while it crossed the nerve twice in only 3 specimens. Mean distance from the radial styloid to the most distal point where the vein crossed the nerve was 5.10 cm.

## Discussion

The sensory branch of the radial nerve emerged between the tendons of the brachioradialis muscle and the long radial extensor muscle of the wrist at a mean distance of 8.54 cm from the styloid process of the radius in all 25 specimens studied. In the study of Abrams et al [7] it reached 11.6 cm and averaged 9.0 cm. Auerbach et al [9] reported similar distances, with an average of 8.6 cm (range, 6.1-11.8 cm). Robson et al [8] reported that the SBRN emerged from under brachioradialis by a mean of 8.31 cm proximal to the radial styloid. Our results are consistent with these studies as the differences were not statistically significant (Table 1)

**Table 1:** comparison of the mean distance of emergence of the SBRN of different studies with calculated P values.

	Mean Distance of emergence of the nerve, from the radial styloid	P value (CI = 95%)
Abrams et al (1992)	9.0 cm	0.46

Auerbach et al (1994)	8.6 cm	0.55

Robson et al (2008)	8.3 cm	0.96

Since P value was > 0.05, differences observed were not statistically significant.

In our study the nerve constantly divided into two branches at a mean distance of 5.6 cm proximal to the radial styloid. Abrams et al [7] observed that the nerve constantly divided into two divisions at a mean distance of 5.1 cm proximal to the radial styloid. Auerbach et al [9] noted that the division occurred at a mean distance of 6 cm proximal to the radial styloid. Robson et al [8] described the division point 4.92 cm proximal to the radial styloid. Calculated P values are given in table 2 which shows that differences observed in different studies were not statistically significant. Thus our data adds another dimension confirming the branching point of the SBRN.

**Table 2:** compares the mean distance of branching point of the SBRN in different studies and the with calculated P values.

Studies	Mean distance of the Division point of the nerve, from the radial styloid	P value (CI = 95%)
Abrams et al (1992)	5.1 cm	0.81

Auerbach et al (1994)	6.0 cm	0.57

Robson et al (2008)	4.9 cm	0.81

Since P value was > 0.05, differences observed were not statistically significant.

In our series, the mean distance to the point where the most medial and most lateral branches of the nerve crossed the wrist joint, as measured from the Lister's tubercle were, 2.51 cm (SD = 0.53) and 3.90 cm (SD = 0.64) respectively. Therefore the risk of damaging the branches of the nerve is particularly high if surgical incisions are placed in that region. This was observed in a study conducted by Robson et al superficial branch of the radial nerve [8] where 25 forearm specimens were dissected and were noted to have branches underlying the traditional transverse incision for de Quervain's release. This finding is also consistent with other cadaveric studies such as Glanvill, R. et al [1], Vandersluis, R. et al [2] where orthoepaedic pin fixation was shown to cause iatrogenic injury to the SBRN, as well as study conducted by Kilic A et al [6] where it was shown that limited size of the area where portals can be positioned and the anatomic variations between individuals are major obstacles in developing a guideline for reducing the risk of SBRN injury in wrist arthroscopy.

In every specimen, we found a large cephalic vein on the lateral aspect of the forearm collecting blood from the superficial venous arch on the back of the hand.

We also considered the cephalic vein-nerve intersections throughout the course of the SBRN. In majority of specimens (68%), we found that vein and nerve intersected at least once, and sometimes even twice. In a study conducted by Robson et al [8] the Cephalic vein intersected the nerve in 80% of specimens.

Our results show that the risk of damaging a nerve when accidentally puncturing the posterior wall of a vein on the lateral or dorsal aspects of the wrist is a constant risk, as the cephalic vein, usually used for punctures, crossed over the sensory branch of the radial nerve in a majority (68%) of specimens. Because the deeper side of the vein and superficial side of the sensory nervous branches are very close to each other, the risk of damaging a nerve is greater if the vein is transpierced at site where the nerve and vein intersect. The highest risk of iatrogenic nerve injury lies at the points of intersection between the nerve and the vein. Since the nerve emerged from beneath the brachioradialis 8.5 cm proximal to the radial styloid, and from that point onwards was closely associated with the nerve, with majority of the specimens (68%) showing at least one intersection, we concluded that the risk of iatrogenic nerve injury was maximal in the lower third of the forearm. Robson, A. J [8] et al also concluded that cannulation of the cephalic vein in the distal third of the forearm is best avoided. Our results help to strengthen the above recommendation.

Limitations of the present study include the fact that we have not compared height and gender of the cadavers that we have dissected, and only absolute measurements with regards to fixed bony points were noted. Therefore we were not in a position to assess whether there was any effect of the height or the gender of the dissected specimens on the results we obtained. Further comparative studies in future are necessary to assess whether there are any differences in the anatomy of the SBRN with regard to height and gender of the individual as well.

## Conclusion

The Variable anatomy of the nerve makes it impossible to define a safe zone for surgical incisions on the dorsum of the wrist. We recommend avoiding transverse incisions in the snuffbox region between 2.5 cm and 3.9 cm from the Listers tubercle as the risk of damaging the branches of the nerve is very high.

We also recommend avoiding cannulation of the cephalic vein in the distal forearm up to 8.5 cm from radial styloid in order to avoid damaging to the nerve.

## List of abbreviations

SBRN: Superficial Branch of Radial nerve

## Competing interests

The authors declare that they have no competing interests.

## Authors' contributions

HL, ST, VB, and LS dissected the specimens and collected the data. LS designed the study, analyzed the data and prepared the manuscript. DA and RJ contributed to revisions of the article and overall supervision of the project. All authors read and approved the final manuscript.
